# European health examination surveys – a tool for collecting objective information about the health of the population

**DOI:** 10.1186/s13690-018-0282-4

**Published:** 2018-06-28

**Authors:** Hanna Tolonen, Päivikki Koponen, Ala’a Al-kerwi, Nada Capkova, Simona Giampaoli, Jennifer Mindell, Laura Paalanen, Maria Ruiz-Castell, Antonia Trichopoulou, Kari Kuulasmaa, for the EHES Network, Günter Diem, Johan van der Heyden, Plamen Dimitrov, Marcel Lappée, Nada Capkova, Allan Linneberg, Anne Christensen, Mare Ruuge, Hanna Tolonen, Ari Haukijärvi, Päivikki Koponen, Kari Kuulasmaa, Laura Paalanen, Tarja Palosaar, Jouko Sundvall, Katja Borodulin, Tiina Laatikainen, Seppo Koskinen, Satu Männistö, Markku Peltonen, Isabelle Gremy, Michel Vernay, Antje Gößwald, Panagiotis Kamtsiuris, Antonia Trichopoulou, Androniki Naska, György Surjan, Arpad Ambrus, Gudrún K. R. Gudfinnsdóttir, Hannah McGree, Karen Morgan, Simona Giampaoli, Luigi Palmieri, Diego Vanuzzo, Iveta Pudule, Vilius Grabauskas, Abdonas Tamasiunas, Jurate Klumbiene, Maria Ruiz-Castell, Ala’a Al-Kerwi, Neville Calleja, Dorothy Gauci, Sarah Cushieri, Dragan Gjorgjev, Monique Verschuren, Grethe S. Tell, Johan Heldal, Susie Jentoft, Wojciech Drygas, Pawel Kurjata, Carlos Dias, Baltazar Nunes, Cristian Calomfirescu, Mária Avdicova, Alenka Borovnicar, Metka Zaletel, Jozica Zakotnik, Monica Saurez Cardona, Anders Tegnell, Banu Ayar, Jennifer Mindell

**Affiliations:** 1 Department of Public Health Solutions, National Institute for Health and Welfare (THL), P.O. Box 30, FI-00271 Helsinki, Finland; 2 Department of Population Health, Luxembourg Institute of Health, Strassen, Luxembourg; 3 Environmental and Population Health Monitoring Centre, National Institute of Public Health, Prague, Czech Republic; 4 Department of Cardiovascular, dysmetabolic and ageing-associated diseases, Istituto Superiore di Sanità (ISS), Rome, Italy; 5 UCL, London, UK; 6 Hellenic Health Foundation, Athens, Greece

**Keywords:** Health information, Survey, Population, Representative, Comparable

## Abstract

**Background:**

Representative and reliable data on health and health determinants of the population and population sub-groups are needed for evidence-informed policy making; planning and evaluation of prevention programmes; and research. Health examination surveys (HESs) including questionnaires, objective health measurements and analysis of biological samples, provide information on many health indicators that are available not at all or less reliably or completely through administrative registers or health interview surveys.

**Methods:**

Standardized cross-sectional HESs were already conducted in the 1980’s and 1990’s, in the framework of the WHO MONICA Project. The methodology was developed and finally, in 2010–2012, a European Health Examination Survey (EHES) Pilot Project was conducted. During this pilot phase, an EHES Coordinating Centre (EHES CC, formerly EHES Reference Centre) was established. Standardized protocols, guidelines and quality control procedures were prepared and tested in 12 countries which conducted pilot surveys, demonstrating the feasibility of standardized HES data collection in the European Union (EU).

Currently, the EHES CC operates at the National Institute for Health and Welfare (THL), Finland. Its activities include maintaining and developing the standardized protocols, guidelines and training programme; maintaining the EHES network; providing professional support for countries planning and organizing their national HESs; external quality assessment for surveys organized in the EU Member States; and development of a centralized database and joint reporting system for HES data.

**Results:**

An increasing number of EU Member States are conducting national HESs, demonstrating a strong need for such surveys as part of the national health monitoring systems. Standardization of the data collection is essential to ensure that HES data are comparable across countries and over time. The work of the EHES CC helps to ensure the quality and comparability of HES data across the EU.

**Conclusions:**

HES data have been used for health monitoring and identifying public health problems; to develop health and prevention programmes; to support health policies and preparation of health-related legislation and regulations; and to develop clinical treatment guidelines and population reference values. HESs have also been utilized to prepare health measurement tools and diagnostic methods; in training and research and to increase health awareness among population.

## Background

Information about health and health determinants at the population level is needed for evidence-informed policy making, planning and evaluation of prevention programmes, and research. Some population level information for the European Core Health Indicators [1, 2], such as mortality, can be obtained from administrative registers but some information has to be collected through health surveys. Health surveys can be classified into health interview surveys (HIS) and health examinations surveys (HES). In HIS, all data is based on self-report and collected through questionnaires, either self-administered or interview-based. In the EU, the European Health Interview Survey (EHIS) is based on EU Regulation [3] and is coordinated by Eurostat.

HESs also always include components from a health interview survey but key data collection is done through objective health measurements and/or analysis of collected biological samples. There is no Regulation about HES in the EU, but many countries have considered HESs to be an important part of their health monitoring system. The *European Health Examination Survey (EHES)* is an initiative, established in 2009, to set up a system of standardized, representative HES in the European countries.

The *Feasibility of a European Health Examination Survey* project was conducted in 2006–2008 to assess the importance of national HESs and the importance and feasibility of their joint standardization [4, 5]. It was followed by the *EHES Pilot Project* in 2009–2012, which prepared survey guidelines and conducted pilot surveys in 12 countries [6, 7]. After the *EHES Pilot Project*, the joint standardization of national HESs and some of the activities of the EHES Coordinating Centre (EHES CC) continued as a part of EUs *BRidging Information and Data Generation for Evidence-based Health policy and research (BRIDGE Health)* project in 2015–2017 [8]. Under the framework of the BRIDGE Health project, the EHES network has been updated; the EHES website has been updated and maintained, and the EHES Manuals together with the related training material have been updated. E-mail consultations for countries planning and organizing their national HES have been provided and some external quality control measures such as site visits and laboratory quality assessment have been conducted.

This article describes the current status of national HESs in EU Member States and how they have been used to enhance public health and health research in countries. Since not all EU Member States have conducted a national HES, key European level actions needed to enhance planning and preparation of standardized national HESs in all the EU Member States are outlined.

## Methods

The information presented in this paper has been collected from the EHES Network members [9] through questionnaires and e-mail correspondence. EHES Network is asked annually to provide updated information about ongoing and planned national HESs in their country. In April 2017, a short questionnaire about supporting actions offered by the EHES CC was sent to the EHES Network [10]. The aim was to find out how useful the national HES organizers considered proposed actions when they are planning and organizing their national HES. Replies were received from 17 persons (41% of recipients of the request). Information about uses of HES data was collected through e-mail correspondence in May–June 2017. Direct contact with the organizers of national HESs was made to learn how collected data have been used in their countries. Some of the information has also been obtained in the face-to-face discussions organized during the site visits by EHES CC members to ongoing national HESs.

## Results

### National HESs in Europe

**Fig. 1:**
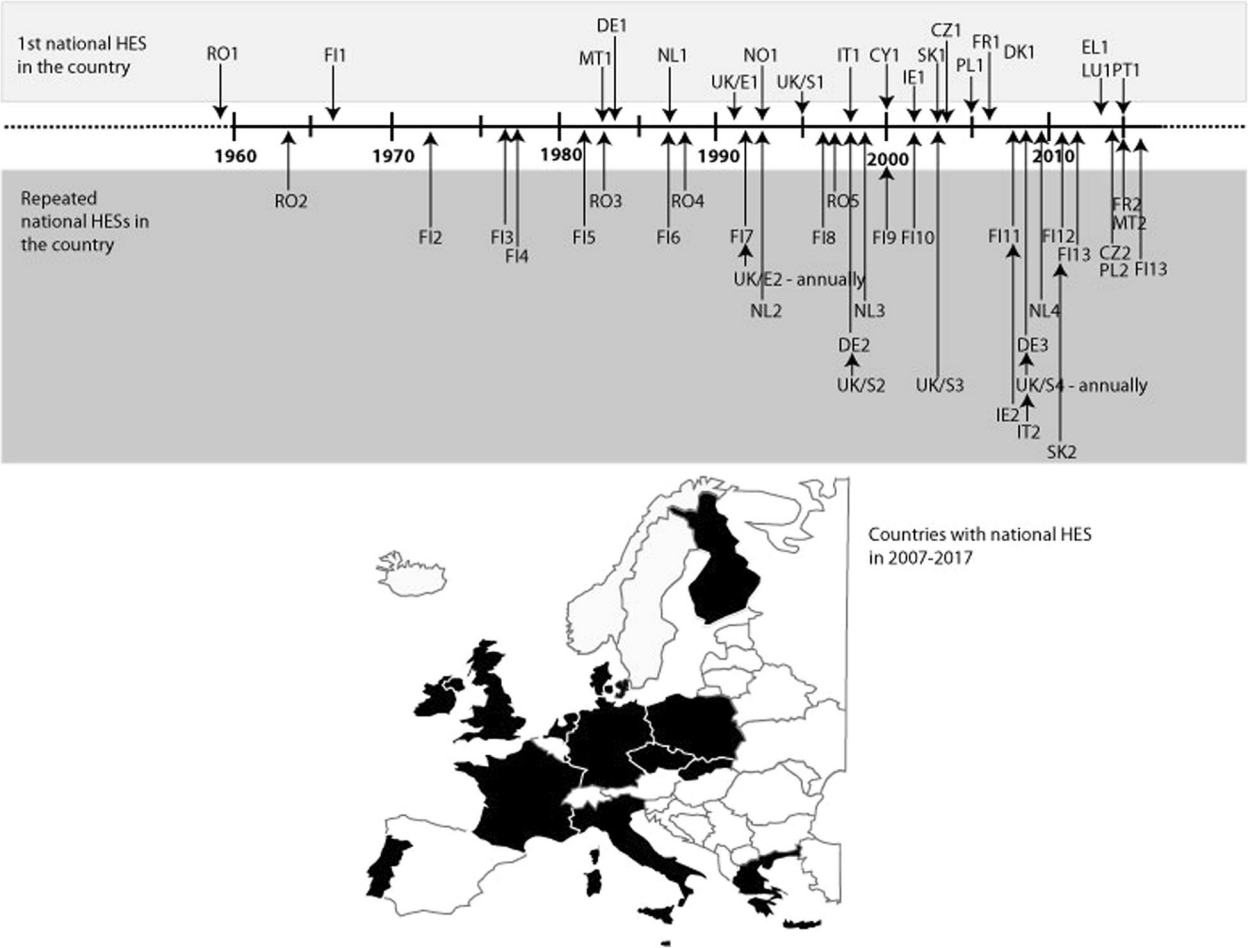
History and current status on national HESs in EU Member States

In Europe, the first national HESs were conducted as early as the late 1950s and early 1960s. These were focused mainly on cardiovascular disease risk factors such as hypertension, blood lipids, smoking and obesity. From the 1970s to the 1990s, there was a steady increase in the number of countries conducting national HESs. Since 2000, there has been a fast increase in new countries conducting a national HES (Fig. 1). At the same time, more measurements and questionnaire modules such as functional capacity, mental health, physical activity and dietary habits have been included in the surveys to provide better information for national health policies [11].

In the past 11 years (2007–2017), national HESs were conducted in 14 countries (Fig. 1), for example FinHealth in Finland, ESTEBAN in France, EHES-LUX and ORISCAV-LUX 1&2 in Luxembourg, INSEF in Portugal, and the Health Survey for England. A detailed list of these surveys is available at the EHES web site (http://www.ehes.info/national/national_hes_status.htm). Most of them followed standardized EHES protocols, ensuring comparability of the core measurements (common key measurements included in every national HES).

Several countries are planning to conduct either their first national HES (e.g. Belgium) or a repeated national HES in 2018–2022 (Czech Republic, Finland, Germany, Portugal and UK/England). Some of these future HESs are planned to be conducted in connection with the European Health Interview Survey (EHIS) which is mandatory in each EU Member State. The third wave of EHIS will take place in 2019.

### Supporting activities of the EHES coordination

With funding from the EU/DG SANTÉ, the EHES Coordinating Centre (formerly called the EHES Reference Centre) was established in 2009 for the EHES Pilot Project and organized jointly by the National Institute for Health and Welfare (THL), Finland (leader); Statistics Norway, Norway; and the Instituto Superiore di Sanitá, Italy. After the EHES Pilot Project ended in 2012, the EHES Coordinating Centre activities were continued on a small scale by THL without external funding until 2015 when some funding was received through the BRIDGE Health Project (2015–2017).

The purpose of the EHES Coordinating Centre is to support countries in planning and organizing their national HES and ensuring that results obtained from these surveys are comparable with other similar surveys conducted in the EU Member States. This includes: development and maintaining of standardized measurement and data collection protocols; guidelines for sampling and for preparing the fieldwork, including training programme for survey organizers and fieldwork staff; providing professional support for countries planning and organizing their national HESs; external quality assessment for surveys organized in EU Member States; and development of a centralized database and joint reporting system for HES data.

### Guidelines and standardized protocols

Guidelines for organizing national HESs have been developed and published as the *EHES Manual, Part A. Planning and preparation of the survey* [12]. It includes guidelines for sampling; legal and ethical issues; selection of questionnaire modules; physical measurements and collected biological samples; data management; recruitment of participants; and preparing the survey budget.

Standardized protocols for core physical measurements and collection of biological samples for measuring cardiovascular disease risk factors have been available since the 1960’s through *Cardiovascular Survey Methods by Rose* [13]. These were further developed by the *WHO MONICA* Project [14], *European Health Risk Monitoring Project (EHRM)* [15], the *European Cardiovascular Surveillance set (EUROCISS)* [16] and *Feasibility of a European Health Examination Survey (FEHES)* Project [4]. The latest European level protocols are available in the *EHES Manual, Part B. Fieldwork procedures* [17] for selected core measurements. The manual covers anthropometric measurements, blood pressure, and blood samples for lipids and glucose. These core measurements provide information about key risk factors for major non-communicable diseases.

A much wider range of physical measurements have been included in several national HESs. [11] These measurements include for example lung function tests, bioimpedance, measurements for cognitive and physical functioning, and extensive dietary interview/questionnaire module. Protocols for urine samples and two core measurements for functional capacity (chair stand and hand grip strength test) have been added to the latest update of the EHES Manual and some other international standardized protocols have been added to the EHES web site. It is important to develop the EHES Manual further by adding standardized protocols for other measurements frequently included in European HESs and covering key public health problems. Meanwhile, providing information about existing international standardized protocols, and listing measurements included in different surveys will help survey organizers to collaborate with each other and enhance the standardization of new measurements in the EU.

### Network

The EHES Network [9] consists of people working on national and/or regional HESs or in organizations which might be responsible for organizing a national HESs in each EU Member State in the future. Currently, all EU Member States are represented in the network; links with national HESs outside EU, such as the National Health and Nutrition Survey (NHANES) in USA, have been established. This network is used to share information and experiences about HESs and related good practices and also to establish research collaborations between national HES organizers via the website and e-mail consultations. Seminars and conferences (face-to-face contacts) enhance exchange of information and build new innovations in collaboration between national HES organizers.

### Professional support

Due to limited resources for the EHES Coordinating Centre, support to the national HES organizers has been mainly limited to e-mail consultations. Survey organizers have sought consultation on such issues as sampling, handling of biological samples, specific measurement protocols and questionnaires. Personal consultation visits either by EHES Coordinating Centre staff or other experienced national HES organizes during the planning of a national HES can be beneficial, since these give possibilities for wider discussions and sharing of problems and potential solutions.

### Quality assurance

Standardized protocols alone do not guarantee high quality, comparable data collection. Therefore, quality assurance procedures such as a training programme and external quality assessment during the data collection are needed. The EHES Coordinating Centre has developed, and maintains, online training material for core measurements and for several topics related to planning a survey [12, 18]. During the EHES Pilot Project, training seminars to train the national trainers were also organized. There is a plan to establish a regular training programme to repeat such training seminars at regular intervals.

External quality assessment procedures [19] include site visits during the survey fieldwork to evaluate the adherence of the standardized protocols, a laboratory quality assessment scheme to evaluate the comparability of cholesterol and glucose measurements between laboratories, and retrospective assessment and documentation of achieved data quality and data collection methods used.

It was demonstrated during the EHES Pilot Project (2009–2012) that standardization of health measurements such as blood pressure between surveys is challenging but possible [20], and that site visits are an important part of the quality assurance procedure [21].

### Data sharing and joint reporting

Analysis of standardized HES data, using common definition of indicators, offers possibilities for comparison between countries and benchmarking. The *EHES Manual, Part C. European level collaboration* [19] provides common definition for key indicators derived from the EHES core measurements and questionnaire module. Many of these indicators are based on the European Community Health Indicators [1, 2].

For effective collaboration in the use of the data from the national HESs, it is important to be able to share individual level survey data easily with research groups in different countries. This facilitates evaluation and documentation of data quality, joint EU-level reporting of health indicators derived from HESs, and use of the data for research.

During the EHES Pilot Project, principles for data sharing and templates for data transfer agreements were drafted, and participating countries provided the EHES Coordinating Centre with a copy of their survey data in a standardized format. An alternativeapproach that could be considered is a distributed database. In a distributed database, the national data from each country would stay in a standardized format in a national server but authorized users could analyse the data from multiple sites as if all the data were virtually located in one central place. Further collaboration is required to find the best solution which fulfils all legal and ethical requirements related to sensitive data; complies with the EU Data Protection directive [22] and the new EU General Data Protection Regulation in force from 25 May 2018 to ensure privacy; and is acceptable for all survey organizers and funders.

Using the data collected during the EHES Pilot Project, it was possible to show large differences in blood pressure profiles between countries [23] and also differences in the extent of under-estimation of obesity, hypertension and high cholesterol by self-reported data [24]. Availability of national level data for similar analysis would enhance benchmarking between countries.

### Needs and expectations of national HES organizers

Proposed supporting actions can be classified into six categories:

- Guidelines and standardized protocols,
- Network of national HES experts,
- Professional support for planning and preparation of a national HES,
- Quality assurance,
- Joint reporting, and
- Experiences on how to use national HES results.

**Fig. 2:**
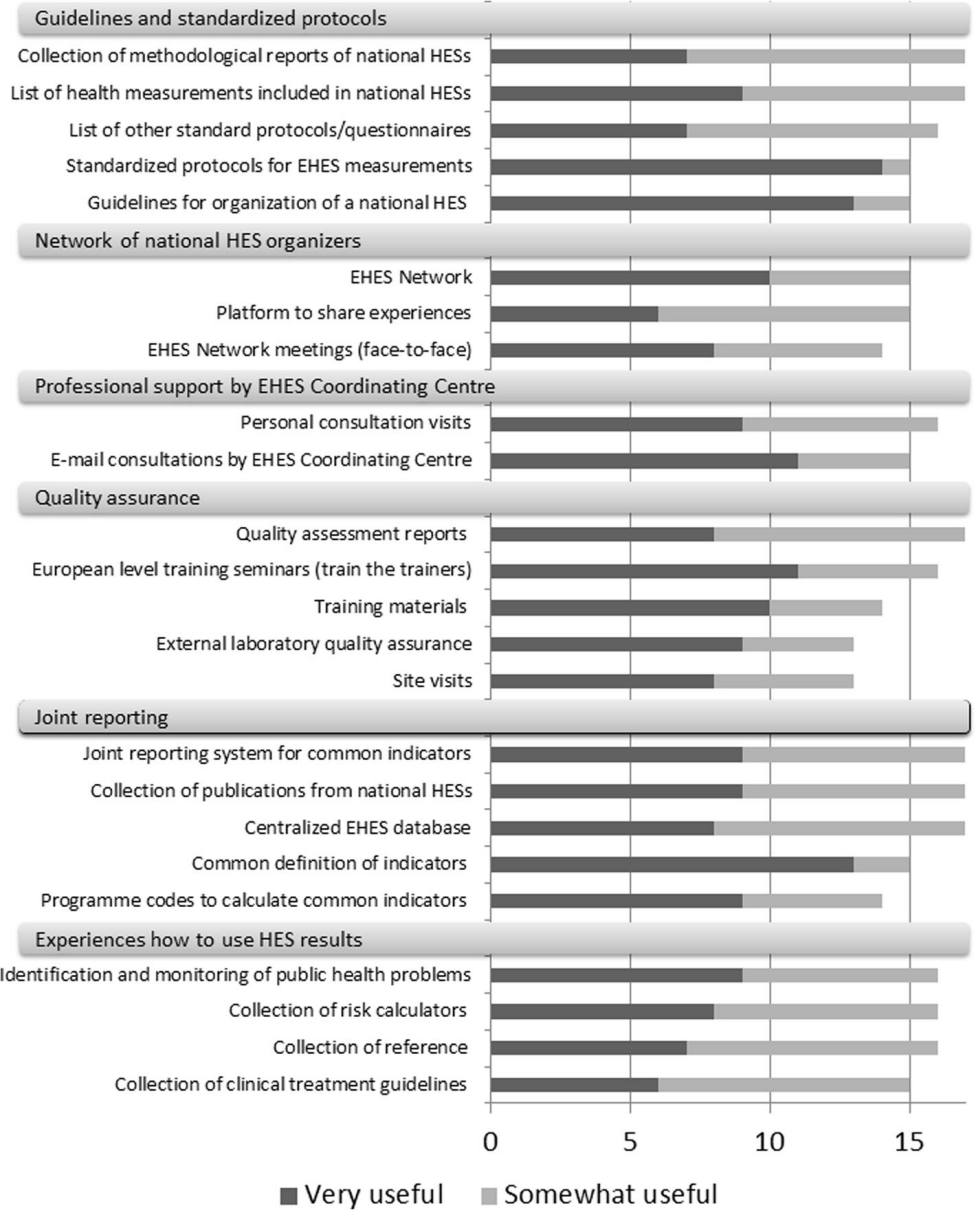
Classification of usefulness of proposed actions to enhance the planning and organization of national HESs

Almost all indicated that the availability of standardized protocols (93%), guidelines for organizing a national HES (87%), and common definitions of indicators (87%) were very useful. Email consultations on specific questions when planning and conducting a national HES were also graded as very useful by 79% of respondents. Training seminars and materials, personal consultations, and programme codes to calculate indicators were found very useful by over 60% of respondents. (Fig. 2.)

### Uses of HES data

**Fig. 3:**
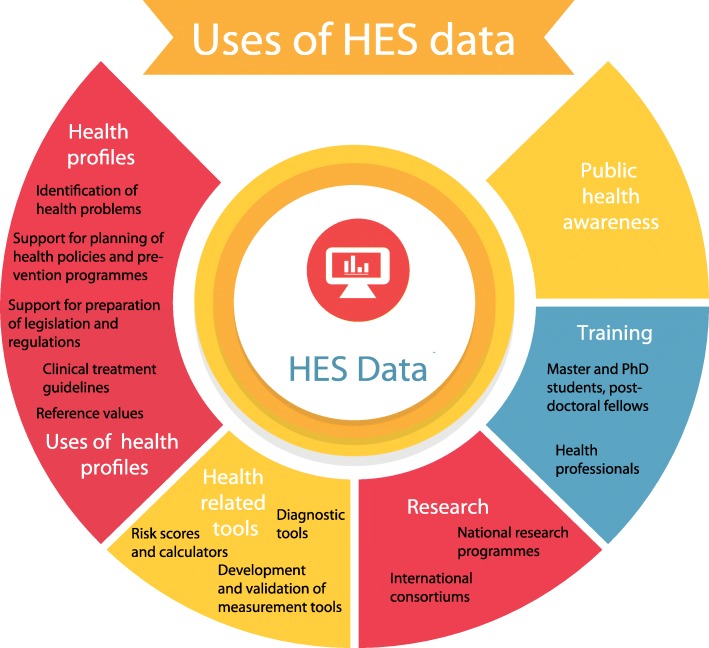
Uses of HES data

Data from national HESs can and have been used widely for health monitoring; to identify public health problems; to develop health and prevention programmes; to support health policies and preparation of health-related legislation and regulations; to develop clinical treatment guidelines and population reference values; to prepare health-related tools and diagnostic methods; in training and research; and to increase health awareness among the population. Some examples from a few European countries have been collected from the EHES network members (Fig. 3).

### Health profiles

National HES data have been used to provide health profiles of the population and population sub-groups and to monitor change in them in the EU Member States. Usually, the health profiles are published on the web, e.g. the Dutch Nederland De Maat Genomen study [25], EHES-survey in the Czech Republic [26], CUORE-Project in Italy [27] and ORISCAV-LUX in Luxembourg [28]. Specific reports or publications have also been prepared in many countries such as Finland [29, 30], Germany [31], Greece [32], the Czech Republic [33–36], the Netherlands [37], Italy [38, 39], Luxembourg [40, 41] and UK/England [42, 43].

The health profiles can be used to identify key health problems in the population or in population sub-groups (e.g. by socio-economic status or ethnic background), in development and evaluation of health policy strategies and programmes and prevention programmes, to support health-related legislation and regulation decision making, in development of clinical treatment and prevention guidelines, and definition of population based reference values.

For example, in Finland and Luxembourg [44], HES data has helped to recognize vitamin D deficiency and in Finland iodine deficiency as well in the population. In Finland this has led to recommendations for supplement use and to fortification of dairy products with vitamin D and salt with iodine. In the Czech Republic, Greece and Luxembourg, HES data have been used to identify risk factor burdens.

Development and evaluation of health policy programmes and strategies has strongly relied on information obtained from national HESs in many countries. For example in Finland and the UK [45], National Obesity Programmes have been based on information about current obesity status from the national HES. Evaluation of these programmes has also relied on national HES data. In Greece, national HES data has been used to identify priorities for public health interventions and in Italy, the Ministry of Health had developed the 2017–2018 National Prevention Plan using the national HES data as background and baseline information. In 2009, the community programme of the Italian Ministry of Health called *“Gaining Health: make healthy choices easy”* signed an agreement with bread-maker associations to reduce salt by 15% in bread in 5 years. National HES data, including a 24-h urine collection, was used to measure salt intake in the diet. Similar data will be collected in the next HES (2018–2019) to measure the changes in salt consumption in the diet of the adult population [46]. In Luxembourg, the national campaign by the Ministry of Health to fight hypertension was promoted by publication of information on prevalence and awareness of hypertension [47].

Decision-making on health-related policies, including setting up new legislation and regulations, requires strong evidence-based support. For example in Finland and the UK [48], information about exposure to second-hand cigarette smoke obtained from national HESs was used to support smoking legislation and also to monitor the effects after the legislation was in force. HES data have also been used to evaluate the potential to reach goals set in the WHO Global Action Plan for Prevention and Monitoring on noncommunicable diseases [49–52]. In Finland, HES data have been widely used as epidemiological background material when preparing national clinical guidelines for obesity, hypertension, dyslipidaemia, smoking cessation, chronic obstructive pulmonary disease (COPD), diabetes and memory disorders [53].

HES data have also been used to identify population level reference values. In Luxembourg, the most suitable definition for the metabolic syndrome for the Europoid population living in Luxembourg was drawn from the ORISCAV-LUX study [54]. More examples of reference values come from the USA, where reference values for dietary intake of dietary supplements, growth charts for children and femur bone mineral density are based on National Health and Nutrition Examination Survey (NHANES).

### Health-related tools

In Finland, several risk scores and calculators (cardiovascular disease (CVD) risk [55], diabetes risk [56] and dementia risk [57]), later widely used also internationally, have also been developed based on HES data. Italy has also prepared national risk charts and scores based on national HES data [58]. The risk scores and calculators are also available on the internet for the general public and are widely used by clinicians in everyday patient consultations. Other potential examples of HES data use for clinical practice include on development of new diagnostic tests. One example is a genetic test for lactose intolerance, which is very common in Finland.

When new measurement tools are developed to be used in HESs or other population studies, they need to be validated. In Luxembourg, the HES was used to validate the Food Frequency Questionnaire (FFQ) among the Luxembourgish population [59] and a national dietary recommendation index has been developed to evaluate diet quality of the adult population [60]. In Greece, the HES was used to validate a digital tool photography atlas use as portion size measurements in dietary surveys [61] and to validate the dietary intake method used in the Greek national health and nutrition survey [62].

### Research

HES data is a rich source for scientific research. Availability of such data has opened possibilities for new national and international research initiatives such as the MORGAM Project [63], CHANCES Project, NCD-Risk Factor Collaboration [64], and the International Global Burden of Disease consortium [65]. National HESs have also served as a baseline for cohort and intervention studies such as diabetes and dementia prevention studies.

Obviously, HES data together with collected and stored biological samples from participants form excellent bases of innovative epidemiological and public health research. National longitudinal studies with re-examination of the participants have also been developed in Finland [66], the Czech Republic [67], Germany [68], Luxembourg [28] and the UK [69]. If there is a possibility to link survey data to administrative registers, cost-effective follow-up of cohorts can be developed based on the cross-sectional national HES. Such cohort and longitudinal studies will provide the opportunity to study trajectories of health and disability.

### Training

HESs are also widely used for training. In Italy, a group of health professionals (cardiologist, nurses, etc.) were trained during the national HES to carry out health measurements in the general population in a standardized way. This has led to awareness of NCD risk factors and potentials for prevention through several community actions of health monitoring in regions. In many countries, HES data have been widely used for Masters and PhD studies as well as among post-doctoral fellows. For example in Finland, with over 40 years tradition of carrying our HESs, over 200 Masters and PhD thesis have been produced based on HES data.

### Public health awareness

In many countries, during Hypertension Day, World Heart Day, Obesity Day, Diabetes Day etc. HES results are presented via different activities to increase awareness of risk factors and prevention among the general population. Collaboration with professional and patient organizations has also been beneficial both for survey planning, fieldwork organization and dissemination of the results.

## Discussion

A HES can provide objective health information, free from reporting and awareness bias. Even though an increasing number of countries are carrying out national HESs, and repeating surveys periodically, about half of the EU Member States have not had a national HES in the past ten years. At the same time, several European countries have identified a need for HESs as a part of their national health monitoring system. The countries and the European Commission (EC) have also indicated the importance of comparability of the HES data between countries and over time. This initiated the EHES process of joint standardization of the national HESs. The required infrastructure was mostly set up during the EHES Pilot Project. Standardized survey protocols were developed, a training programme was prepared, a quality assessment system together with data management and reporting systems were established and possibilities for advice through consultations were built.

Regardless of the widely recognised need for cross-national standardization of national HESs, past and current actions have been based on temporary solutions through project-based funding. While it is mandatory for EU Member States to organize a national health interview survey (the EHIS), EHES does not have a similar legal status. Countries which have conducted a national HES have done that based on their national health monitoring and research needs. A final decision to conduct a national HES is often dependent on funding being available. This has put countries in very unequal positions in relation to availability of objective health information. Even though methods to conduct a standardized national HESs exist, final decisions rely on national policy makers. Do they see the need for objective health information on the general population at the national level, and are they willing to invest in that?

The challenge now, when the system has been set up and countries are strongly relying on it, is to develop EHES into a sustainable system. A sustainable EHES Coordinating Centre would support countries in conducting periodic HESs and would help when setting up national HESs in countries without past experience of these. If we let the system that has been set up run down, individual countries may not pay attention to standardization and comparability, preventing European level comparisons and benchmarking in the future.

Establishing a European Research Infrastructure Consortium (ERIC) on Health Information has been discussed. This could be one option to ensure sustainability of EHES activities, if the ERIC can be set up in the next few years and a large number of EU Member States are committed to it. Both at the national and European level, it should also be noted that a national HES can be conducted in connection with: a national health interview survey such as the European Health Interview Survey [3]; dietary surveys proposed by EFSA [70]; human biomonitoring surveys, such as ones proposed under the European Human Biomonitoring Initiative (HBM4EU) [71]; or serve as bases for more specific studies such as communicable disease studies planned by the European Centre for Disease Prevention and Control (ECDC) [72].

It has been also raised up occasionnally whether could the coordination of national HESs could be done by Eurostat or ECDC. HES is a survey, as is EHIS, and therefore in principle it could fit within Eurostat. Eurostat is the statistical authority with the legal mandate to collect information for European statistical purposes [73]. Their data collections are based on regulations; since national HESs do not currently have a legal basis in the EU, it is not possible for them to be coordinated by Eurostat. There is similar situation is with ECDC, whose mandate is related to communicable diseases by EU regulation [74]. Therefore, establishing the coordination of national HESs under Eurostat or ECDC would require new regulation which is currently not foreseen.

As demonstrated above with country examples of uses of HES data, the surveys have an important role in public health policy, practice, research and professional training. Many health professionals are using in their everyday practice, tools and tests developed based on HES data. Over the years, hundreds of public health professionals have obtained their qualifications using HES data in their Master and PhD thesis or during post-doctoral fellowships.

The research potential of HES data on public health and epidemiological research are wide, especially when some of the collected biological samples (blood, urine, DNA, etc.) are stored for future use in biobanks. They can help to identify new biomarkers and risk factors for existing public health problems but also identify new, potential public health problems and their risk factors. Stored biological samples provide material to investigate and develop new diagnostic methods and treatments.

The value of HES data for research has increased substantially by the possibility of linking HES data with administrative registers in many countries. A common example is the linkage of HES data with death registers (for mortality and causes), and increasingly also with hospital discharge registers, outpatient registers and drug prescription registers. Such record linkage enables establishing cohorts where the survey data provides a baseline and mortality and morbidity follow-up is conducted through data linkage. This is a cost-effective study design which usually has very good coverage. The possibility to link HES data and biobanks with administrative registers has made HES data a part of Big Data, which has a major potential for health information and research in future.

## Conclusions

Data from national HESs is valuable for health monitoring, planning and evaluation of public health policies and prevention programmes, research, and professional training. It also helps to develop tools and practices used in health care. Therefore, an investment to a national HES will provide many fold profit for society, especially for public health.

## References

[1] ECHI - European Core Health Indicators. 2017 [cited 2017 10 May]; Available from: http://ec.europa.eu/health/indicators/indicators_en.

[2] Verschuuren M (2013). Public health indicators for the EU: the joint action for ECHIM (European Community health indicators & monitoring). Arch Public Health.

[3] Regulation (EC) No 1338/2008 of the European Parliament and of the Council of 16 December 2008 on Community statistics on public health and health and safety at work. Official Journal of the European Union. 31.12.2008. L354/70. Available at: https://eur-lex.europa.eu/legal-content/EN/TXT/?uri=CELEX:32008R1338.

[4] Tolonen, H., et al., Recommendations for the Health Examination Surveys in Europe. Vol. B21/2008. 2008. Available at: http://urn.fi/URN:ISBN:978-951-740-838-7, Helsinki: National Public Health Institute.

[5] Tolonen, H., et al., Review of Health Examination Surveys in Europe. Vol. B18/2008. 2008. Available at: http://urn.fi/URN:ISBN:978-951-740-843-1, Helsinki: National Public Health Institute.

[6] Kuulasmaa K (2012). An overview of the European health examination survey pilot joint action. Arch Public Health.

[7] Tolonen H (2014). European health examination survey--towards a sustainable monitoring system. Eur J Pub Health.

[8] BRIDGE Health Project. BRIDGE Health. BRidging Information and Data Generation for Evidence-based Health Policy and Research. 2017 [cited 2017 18 September]; Available from: http://www.bridge-health.eu.

[9] EHES Network 2017 [cited 2017 17 November]; Available from: http://www.ehes.info/contact/contact_countries.htm.

[10] Tolonen H, Koponen P, Kuulasmaa K (2017). Blueprint for actions to enhance the organization of national HESs in the EU member states.

[11] Tolonen H (2017). Inequalities in availability of health information from national health examination surveys in EU Member States.

[12] Tolonen, H., ed. EHES Manual*:* Part A. Planning and preparing of the survey. 2nd edition*.* 2016. Available at: http://urn.fi/URN:ISBN:978-952-302-700-8, National Institute for Health and Welfare. Directions 2016_013: Helsinki.

[13] Rose GA, Blackburn H (1968). Cardiovascular survey methods. Monograph Series World Health Organization.

[14] Tunstall-Pedoe, H. and For the WHO MONICA project, eds. MONICA Monograph and multimedia sourcebook. World’s largest study on heart disease, stroke, risk factors, and Popul Trends 1979*–*2002. 2003, World Health Organization: Geneva.

[15] Tolonen H (2002). Recommendation for indicators, international collaboration, protocol and manual of operations for chronic disease risk factor surveys.

[16] Primatesta P (2007). Cardiovascular surveys: manual of operations. Eur J Cardiovasc Prev Rehabil.

[17] Tolonen, H., ed. EHES Manual: Part B. Fieldwork procedures*.* 2nd edition*.* 2016. Available at: http://urn.fi/URN:ISBN:978-952-302-701-5, National Institute for Health and Welfare. Directions 2016_014: Helsinki.

[18] EHES. EHES Training materials. 2012 [cited; Available from: http://www.ehes.info/training_materials/index.htm.

[19] Tolonen, H., ed. EHES Manual: Part C. European level collaboration*.* 2nd edition*.* 2016. Available at http://urn.fi/URN:ISBN:978-952-302-702-2, National Institute for Health and Welfare. Directions 2016_015: Helsinki.

[20] Tolonen H (2015). Challenges in standardization of blood pressure measurement at the population level. BMC Med Res Methodol.

[21] Tolonen H, et al. Standardization of physical measurements in European health examination surveys-experiences from the site visits. Eur J Pub Health. 2017; 10.1093/eurpub/ckw271 28115418

[22] Regulation (EU) 2016/679 of the European Parliament and of the Council on 27 April 2016 on the protection of natural persons with regard to the processing of personal data and on the free movement of such data, and repealing Directive 95/46/EC (General Data Protection Regulation). 2016.

[23] Tolonen H (2016). Blood pressure profiles, and awareness and treatment of hypertension in Europe - results from the EHES pilot project. Public Health.

[24] Tolonen H (2014). Under-estimation of obesity, hypertension and high cholesterol by self-reported data: comparison of self-reported information and objective measures from health examination surveys. Eur J Pub Health.

[25] RIVM. Resultaten in detail. 2012 [cited; Available from: https://www.rivm.nl/Onderwerpen/N/Nederland_de_Maat_Genomen/Resultaten_in_detail.

[26] Státní Zdravotní Ústav (2017). Zdravotní stav ceské populace - výsledky studie EHES 2017.

[27] Istituto Superiore di Sanità. Il Progetto Cuore. 2017 [cited 2017 15 August]; Available from: http://www.cuore.iss.it.

[28] Luxembourg Institute of Health. ORISCAV-LUX. Observation des Risques et de la Santé Cardiovasculaire au Luxembourg. 2017 [cited 2017 15 August]; Available from: http://www.oriscav.lih.lu.

[29] Borodulin K, et al. Kansallinen FINRISKI 2012 -terveystutkimus - Osa 2: Tutkimuksen taulukkoliite: National Institute for Health and Welfare, Report 2013_022_Osa_II; 2013. Available at: http://urn.fi/URN:ISBN:978-952-302-054-2

[30] Koskinen S, Lundqvist A, Riskiluoma N. Terveys, toimintakyky ja hyvinvointi Suomessa 2011: National institute for health and welfare, Report 2012_069; 2012. Available at: http://urn.fi/URN:ISBN:978-952-245-769-1

[31] Robert Koch Institute. *DEGS1: Results*. 2013 [cited; Available from: https://www.rki.de/EN/Content/Health_Monitoring/HealthSurveys/Degs/DEGS1_results/DEGS1_results_node.html.

[32] HYDRIA Project, HYDRIA (2017). Nutrition and Health of the Population in Greece. Findings, Conclusions and Proporsals for Policy Actions.

[33] Capkova N (2016). Health status of the Czech population: results from EHES 2014.

[34] Capkova N (2017). Selected health indicators in the Czech popualtion - EHES study 2014. Hygiena.

[35] Lustigova M, Capkova N (2017). Prevalence of cardiovascular risk factors in the Czech Republic in demographic characteristics - selected results of the EHES study. Demografie.

[36] Zejglicova K (2017). Selected health indicators in the Czech population - EHES study 2014. Prakticky lekar.

[37] Blokstra, A., et al., Nederland de Maat Genomen, 2009–2010. Monitoring van risicofactoren in de algemene bevolking. 2011, RIVM Report 260152001/2011. Available at: https://www.rivm.nl/Documenten_en_publicaties/Wetenschappelijk/Rapporten/2012/januari/Nederland_de_Maat_Genomen_2009_2010_Monitoring_van_risicofactoren_in_de_algemene_bevolking.

[38] Giampaoli S, Vanuzzo D (2014). La salute cardiovascolare degli italiani. Terzo Atlante Italiano della Malattie Cardiovascolari. Edzione 2014. G Ital Cardiol.

[39] Giampaoli S (2015). Cardiovascular health in Italy. Ten-year surveillance of cardiovascular diseases and risk factors: Osservatorio Epidemiologico Cardiovascolare/health examination survey 1998-2012. Eur J Prev Cardiol.

[40] Alkerwi A (2010). First nationwide survey on cardiovascular risk factors in grand-duchy of Luxembourg (ORISCAV-LUX). BMC Public Health.

[41] Alkerwi A (2012). La situation épidémiologique des facteurs de risque cardiovasculaire potentiellement modifiables chez les adultes résidant au Luxembourg, en 2007-2008. Résultats de l’étude ORISCAV-LUX “Observation des Risques et de la Santé Cardiovasculaire au Luxembourg”, in Bulletin Luxembourgeois de la recherche et des études en santé publique.

[42] UCL. Health Survey for England (HSE). 2017 [cited; Available from: https://www.ucl.ac.uk/hssrg/studies/hse.

[43] Fuller E, et al. Health survey for England 2015: health*,* social care and lifestyles: NHS Degital: Leeds; 2016. Available from http://content.digital.nhs.uk/pubs/hse2015.

[44] Alkerwi A (2015). Prevalence and correlates of vitamin D deficiency and insufficiency in Luxembourg adults: evidence from the observation of cardiovascular risk factors (ORISCAV-LUX) study. Nutrients.

[45] Oyebode O, Mindell J (2013). Use of data from the health survey for England in obesity policy making and monitoring. Obes Rev.

[46] Strazzullo P (2012). Population based strategy for dietary salt intake reduction: Italian initiatives in the European framework. Nutr Metab Cardiovasc Dis.

[47] Ruiz-Castell M (2016). Hypertension burden in Luxembourg: individual risk factors and geographic variations, 2013 to 2015 European health examination survey. Medicine (Baltimore).

[48] Oyebode O, Mindell JS (2014). A review of the use of health examination data from the health survey for England in government policy development and implementation. Arch Public Health.

[49] Peltonen M, et al. WHO aims to stop the increase of obesity and type 2 diabetes - action is needed in Finland, in Data Brief *2015_027*: National Institute for Health and Welfare; 2015. Available from http://urn.fi/URN:ISBN:978-952-302-513-4

[50] Borodulin K, et al. The public health goals of WHO for increasing physical activity are achievable, in Data Brief *2015_023*: National Institute for Health and Welfare; 2015. Available from http://urn.fi/URN:ISBN:978-952-302-509-7

[51] Vartiainen E, et al. The target of WHO to lower the mortality rate is achievable - but the requisite is to lower the level of serum cholesterol, in Data Brief *2015_026*: National Institute for Health and Welfare; 2015. Available from http://urn.fi/URN:ISBN:978-952-302-512-7

[52] Laatikainen T, Jula A, Jousilahti P. The target set by WHO to reduce blood pressure will not be rearched without nutritional changes and more effective care, in Data Brief 2015_025: National Institute for health and welfare; 2015. Available from http://urn.fi/URN:ISBN:978-952-302-511-0

[53] DUODECIM. Current Care Guidelines. Guideline Abstracts. 2017 [cited 2017 15 August]; Available from: http://www.kaypahoito.fi/web/english/guidelineabstracts.

[54] Alkerwi A (2011). Prevalence of the metabolic syndrome in Luxembourg according to the joint interim statement definition estimated from the ORISCAV-LUX study. BMC Public Health.

[55] National Institute for Health and Welfare. RINRISK-calcutor. 2017 [cited 2017 15 August]; Available from: https://www.thl.fi/en/web/chronic-diseases/cardiovascular-diseases/finrisk-calculator.

[56] Association, F.D. Type 2 diabeteres risk assessment form. 2017 [cited 2017 15 August]; Available from: http://www.diabetes.fi/files/502/eRiskitestilokeme.pdf.

[57] Muistiliitto. Riskitesti. 2017 [cited 2017 15 August]; Available from: https://www.facebook.com/muistiliitto/app/114770661946535.

[58] Istituto Superiore di Sanità. Cuore.exe. 2017 [cited 2017 15 August]; Available from: http://www.cuore.iss.it/cuore_exe/cuore_exe.asp.

[59] Sauvageot N (2013). Use of food frequency questionnaire to assess relationships between dietary habits and cardiovascular risk factors in NESCAV study: validation with biomarkers. Nutr J.

[60] Alkerwi A (2012). Population compliance with national dietary recommendations and its determinants: findings from the ORISCAV-LUX study. Br J Nutr.

[61] Naska A (2016). Evaluation of a digital food photography atlas used as portion size measurement aid in dietary surveys in Greece. Public Health Nutr.

[62] Hellenic Health Foundation. HYDRIA survey. 2017 [cited 2017 14 August]; Available from: http://www.hhf-greece.gr/hydria-nhns.gr/index_eng.html.

[63] Evans A (2005). MORGAM (an international pooling of cardiovascular cohorts). Int J Epidemiol.

[64] NCD Risk Collaboration. NCD RisC. 2017 [cited 2017 15 August]; Available from: http://www.ncdrisc.org.

[65] Institute for Health Metrics and Evaluation. Global Burden of Disease (GBD). 2017 [cited 2017 15 August]; Available from: http://healthdata.org/gbd.

[66] Lundqvist A, Mäki-Opas T. Health 2011 Survey - Methods: National Institute for Health and Welfare, Report 2016_008; 2016. Available at: http://urn.fi/URN:ISBN:978-952-302-669-8

[67] Zejglicova K (2016). Trends in health indicators in the urban middle-aged population in the Czech Republic in 1998-2010. Public Health.

[68] Scheidt-Nave C (2012). German health interview and examination survey for adults (DEGS) - design, objectives and implementation of the first data collection wave. BMC Public Health.

[69] Steptoe A (2013). Cohort profile: the English longitudinal study of ageing. Int J Epidemiol.

[70] European Food Safety Authority, Guidance of EFSA (2014). Guidance on te EU menu methodology. EFSA J.

[71] HBM4EU. HBM4EU. 2017 [cited 2017 14 August]; Available from: http://www.hbm4eu.eu.

[72] European Centre for Disease Prevention and Control. The development of a seroprevalence survey for hepatitis C in EU/EEA countries. 2016 [cited 2017 14 August]; Available from: https://ecdc.europa.eu/en/about-us/procurement-and-grants/development-seroprevalence-survey-hepatitis-c-eueea-countries.

[73] Eurostat. Overview. 2017 [cited 2017 17 November]; Available from: http://ec.europa.eu/eurostat/about/overview.

[74] Regulation (EC) No 851/2004 of the European Parliament and of the Council of 21 April 2004 establishing a European centre for disease prevention and control. Official Journal of the European Union. 30.4.2004. L142/1. Available at: https://eur-lex.europa.eu/legal-content/EN/ALL/?uri=CELEX%3A32004R0851.

